# A Novel Tram Stent Method in the Treatment of Coronary Bifurcation Lesions – Finite Element Study

**DOI:** 10.1371/journal.pone.0149838

**Published:** 2016-03-03

**Authors:** Mark C. Arokiaraj, Gianluca De Santis, Matthieu De Beule, Igor F. Palacios

**Affiliations:** 1 Cardiology, Pondicherry Institute of Medical Sciences, Pondicherry, India; 2 FEops nv, Technologiepark 3, Ghent, Belgium; 3 Cardiology, Massachusetts General Hospital, Boston, Massachusetts, United States of America; University of Buenos Aires, Faculty of Medicine. Cardiovascular Pathophysiology Institute., ARGENTINA

## Abstract

A novel stent was designed for the treatment of coronary bifurcation lesion, and it was investigated for its performance by finite element analysis. This study was performed in search of a novel method of treatment of bifurcation lesion with provisional stenting. A bifurcation model was created with the proximal vessel of 3.2 mm diameter, and the distal vessel after the side branch (2.3 mm) was 2.7 mm. A novel stent was designed with connection links that had a profile of a tram. Laser cutting and shape setting of the stent was performed, and thereafter it was crimped and deployed over a balloon. The contact pressure, stresses on the arterial wall, stresses on the stent, the maximal principal log strain of the main artery and the side-branch were studied. The study was performed in Abaqus, Simulia. The stresses on the main branch and the distal branch were minimally increased after deployment of this novel stent. The side branch was preserved, and the stresses on the side branch were lesser; and at the confluence of bifurcation on either side of the side branch origin the von-Mises stress was marginally increased. The stresses and strain at the bifurcation were significantly lesser than the stresses and strain of the currently existing techniques used in the treatment of bifurcation lesions though the study was primarily focused only on the utility of the new technology. There is a potential for a novel Tram-stent method in the treatment of coronary bifurcation lesions.

## Introduction

Bifurcation lesions in the coronary arteries are commonly seen in clinical practice [1–5]. The bifurcation lesions can be classified based on anatomic variations [4–6]. Frequently the side branches in bifurcation have significant lesions, which need to be stented. There are many techniques that are available for treatment of these types of lesions. The provisional stent technique, crush and mini-crush are some of the techniques commonly performed techniques for these lesions [7–19]. These methods are complex, time-consuming, and require more coronary hardware and contrast. Also, the complication rates are higher in these conditions. Many clinical studies and pooled analysis from Nordic and British Bifurcation Coronary (BBC) studies, which studied bifurcation treatment procedures have reported a high composite end points of about 18% for these procedures, especially for the 2 stent technique, and about 10% with one stent technique [20–27]. Bench studies also report high stress levels in the main branch using 2 stent approach as well as distortions in the distal stent irrespective of the stent designs during balloon manipulations in the side branch [28–31]. Therefore, these procedures are not comfortably performed in routine practice, and more difficulties would be encountered in the setting of acute coronary syndromes especially after acute myocardial infarctions. Also, most interventional cardiologists are not at ease in performing this procedure due to its complexity. We investigated a novel coronary bifurcation technique, primarily to simplify the treatment method for the same.

## Methods

### Development of tram design and crimping

**Fig 1:**
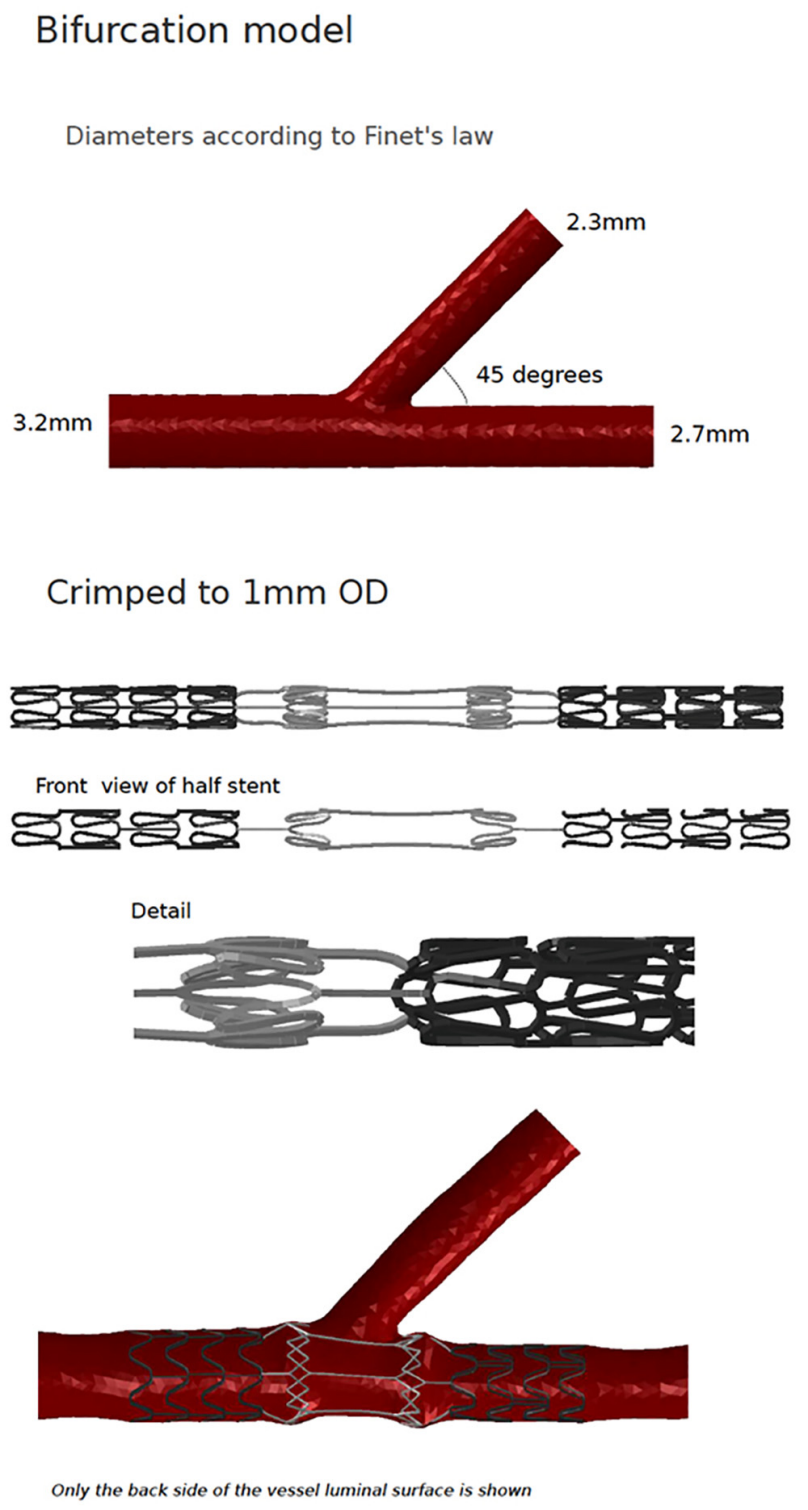
The creation of bifurcation model with side branch origin at 45 degrees, crimping of the stent and deployment of the stent in the bifurcation model.

A novel stent was designed with an interface of nitinol-based 3 connection links interposed in the stent, which is to be placed at the origin of the side-branch. The side-branch was modeled with a diameter of 2.3 mm, and it was placed in the middle of the main branch’s stent. The stent has 2 cobalt chromium parts on either side of the nitinol connections, which are of standard cut design. The strut dimensions of the stent were 70/70 μm (thickness/width). The nitinol connections also had strut dimensions of 70/70 μm. The stent could be crimped and mounted on a balloon. The crimp profile for the stent was 1.0 mm, outside diameter. The crimped stent was mounted over a balloon and deployed in a bifurcation model chosen as shown in Fig 1. The bifurcation model was built based on the Finet’s law, and the main branch and the side-branch profiles were derived from the fractal value of 0.68[32]. Post-dilatation of the stent was not performed.

### Stage 1 finite element analysis

**Fig 2:**
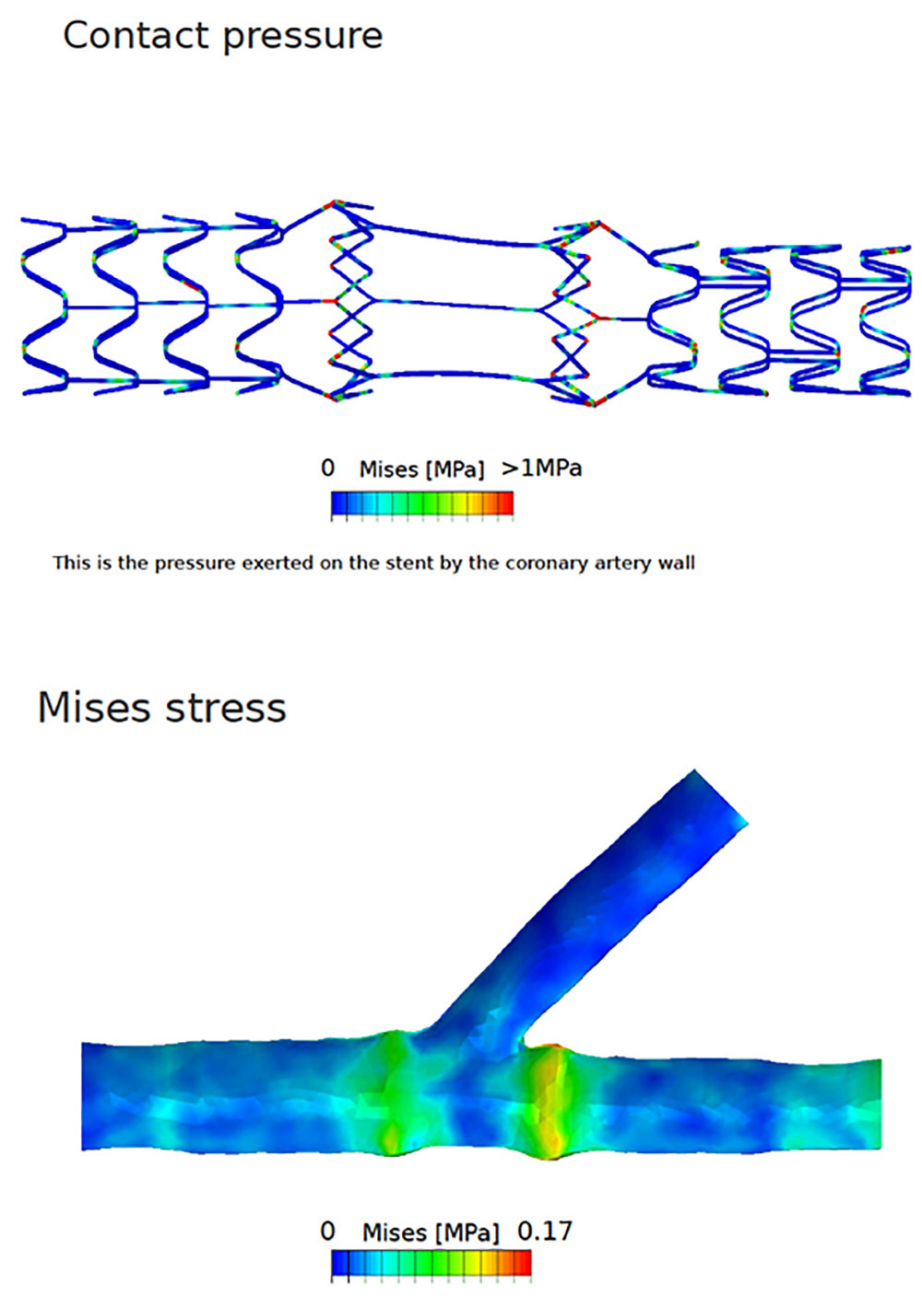
Stage 1 result of the finite element simulations—Stresses on the stent (upper panel) and stresses on the arterial wall (lower panel).

Stage 1 finite element analysis was performed after deployment of the stent. The stent had the minimal increase in stresses on either side of the side branch (Fig 2). However, the rise in the stress was not higher than the stresses observed in other conventional methods for bifurcation lesions. Finite element analysis is now used for evaluation of performance of coronary stents, and it has been used in the evaluation of bifurcation stent evaluation in virtual studies [33, 34].

### Material properties of stent

The Cobalt-Chromium alloy was modeled using the elastoplastic material parameters reported by Clerc and colleagues [35]. The superelastic material model for Nitinol was adapted from Gong et al. [36]. As already described in Iannaccone et al. [37]: the stress–strain relationship of the nitinol alloy (Ti–55.8 wt% Ni) was retrieved from literature, [36] and defined using an embedded user subroutine in Abaqus based on the model of Auricchio et al. [38] Symmetry in compression was assumed (S1 Table). All phases of the device manipulation were assumed to occur at body temperature of 37°C, and Nitinol properties were adapted from Iannaccone et al. [37] (S1 Table). It is important to notice that the security of the welding between Nitinol-elastoplastic metal should not be an issue because in the proposed design the number of welding connection is limited (3 or 4 around the circumference) and, as a result, the cross-sectional size of the connection can be chosen as large as it is needed for stability without impacting the crimpability.

### Material properties of coronary bifurcation model

The layered structure of the arterial wall was taken into account, and different isotropic hyperelastic material properties were assigned to the different tissue layers (intima, media, and adventitia) [39, 40] and the plaques [41]. All simulations were performed using the Abaqus/Explicit finite element solver (Dassault Systèmes, Velizy, France). The ratios of adventitia, media, and intima thickness to total wall thickness were 39%, 35% and 26%, respectively, as reported by Holzapfel et al [39].

### FEA methodology

The coronary artery with a plaque was discretized using multi-block structured conformal hexahedral mesh of 16470 C3D8R elements (4410 in the adventitia, 4410 in the media, 4410 in the intima and 3240 in the plaque), using the methodology described De Santis et al. 2010 [42]. Each of three Cobalt-Chromium stents was modeled using 438 beam elements of type B31 whereas the nitinol stent was modeled using 384 beam elements of type B31. The mesh beam resolution was chosen in order to balance the geometrical shape and the ratio between beam length and cross-sectional dimensions, as reported by De Bock and collaborators [43]. Each beam element was associated to a square cross-section oriented according to user-defined directions. We have chosen the mesh based on similar published studies. For example, in our previous work by Mortier et al. [44], the coronary bifurcation model included 12000 elements for similar artery dimensions (length and diameters) and three-layered wall. For safety concerns, we have increased by 40% the number of elements in the mesh as compared to Mortier et al. [44], including 16470 hexahedral elements.

**Fig 3:**
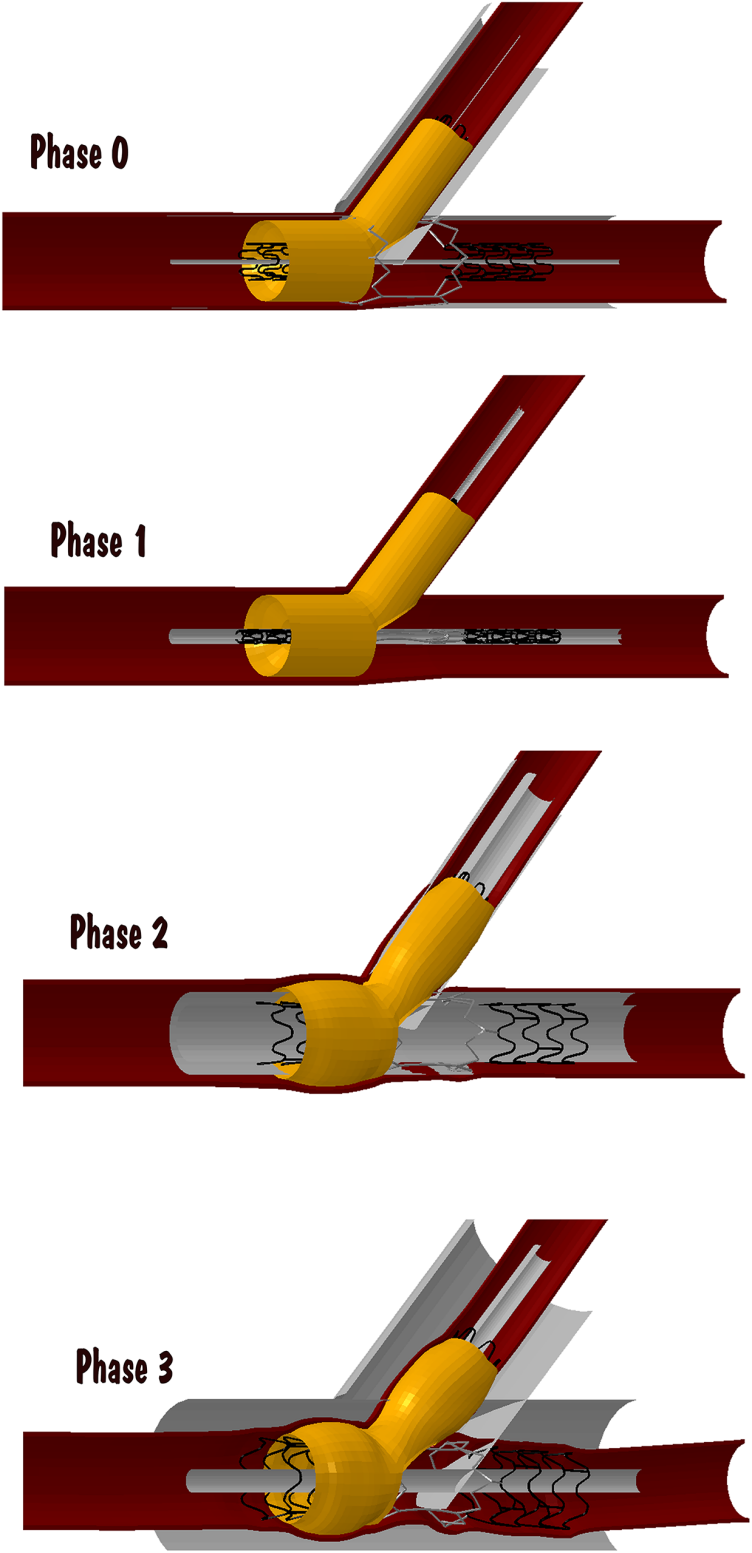
Describes the phases (phase 0 to 3) of deployment of the stent. Description of details of deployment given in text.

To deploy the superelastic parts and elastoplastic parts a procedure described in the previous study, [40] and the procedure described in the past studies [45,46] were merged into a single simulation. For the simulation with two stents, two sets of rigid cylinders, one on the outer side of the stent (CO) and one on the inner side of the stent (CI) have been used in a simulated made of 3 phases as described in Fig 3. In phase 0, the stents are positioned in the bifurcation region with the two sets of cylinders. At phase1 deployment, the stents are crimped by reducing the diameters of the outer cylinders. During phase 2, the outer cylinders are expanded until no contact occurs for the rest of the simulation. The inner cylinders are expanded to plastically deform the elastoplastic part of the stents. The inner cylinder expansion reproduces the balloon expansion. Finally in the phase 3 deployment, the inner cylinders narrow until no contact occurs for the rest of the simulation. At this point, the system is in equilibrium and represents the post-interventional scenario. During the explicit simulation, the kinetic energy was monitored, and the vibrations were visually evaluated to ensure the quasi-static assumption. For the settings of simulation, we refer to the study by Auricchio et al. [38].

### Stage 2, evaluation and bifurcation model with lesions

**Fig 4:**
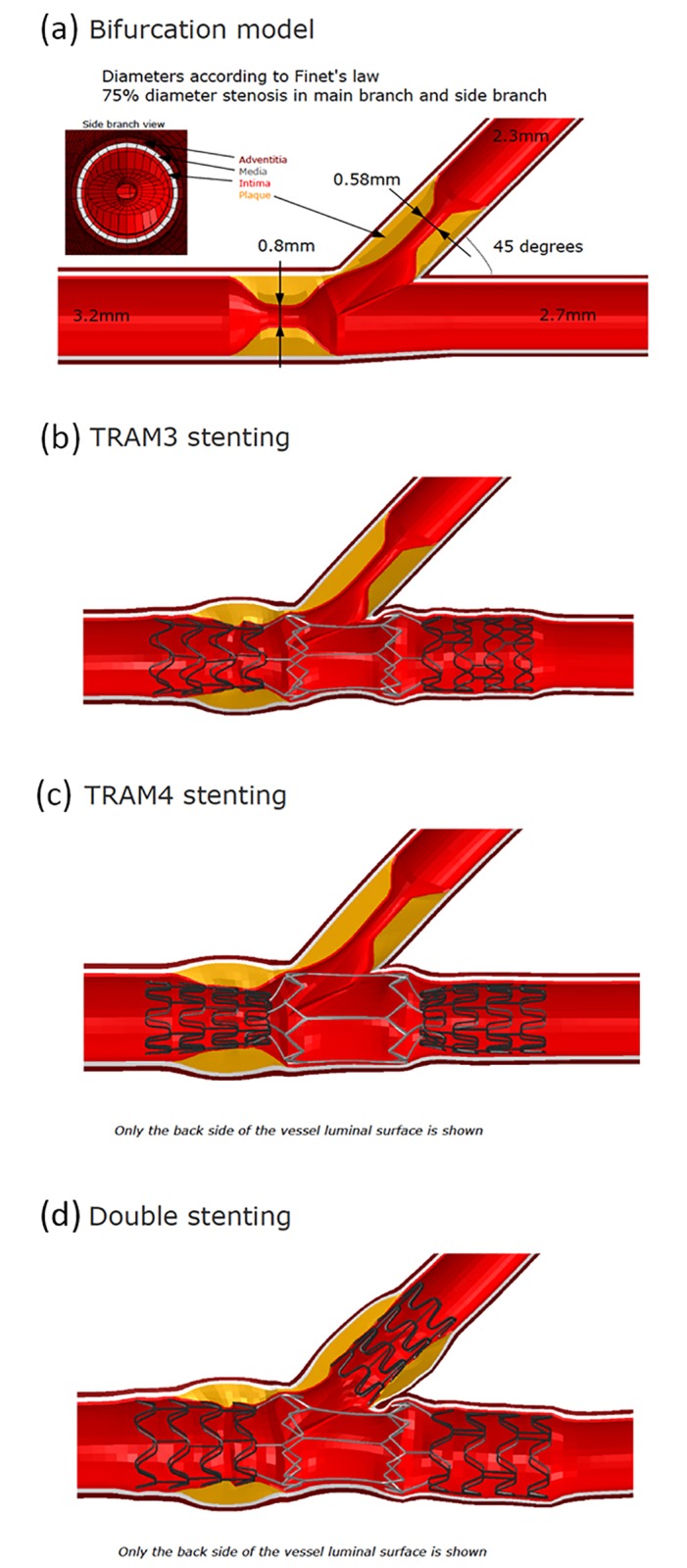
Stage 2 of the study which shows (a) the bifurcation model with the lesion (b) the disposition of the deployed stent with 3 connecting links (c) the disposition of the deployed stent with 4 connecting links (d) Double stenting method.

In the stage 2 of the study, a bifurcation model was created with 2 firm lesions. The first lesion was in the main branch, and the second lesion was in the ostium of the side branch (Fig 4a). Both lesions were modeled to be firm, and the degree of diameter stenosis was about 75%.

### Final tram design development and parametric study

Subsequently, the connection links were modified as 3 or 4 connecting links made of nitinol (Fig 4). This is primarily to test the ability of the stent performance by 3 or 4 nitinol connecting links in the tram area. Since during deployment of the side branch stent if a connecting strut is cut, it would end with 2 struts though the likelihood of complete strut breakage is significantly less. Also many parametric designs were tested in the tram area. Though other designs were parametrically tested it was difficult to effectively crimp on the balloon at 1mm, outside diameter crimp profile. Hence, it was challenging and several designs were developed in the process, for example, connecting rings in the tram connection links; and they were checked for effective crimping profile over the balloon. First crimping of the model was tested and the model would be developed only after its ability to be crimped. Hence, the tram was developed as a final design in our design analysis. The dimension of the nitinol part or the tram area was 20% larger than the cobalt chromium part of the stent in the stage 1 study. This was subsequently reduced to 10% in the stage 2 analyses. This is mainly to evaluate the load bearing capacity of the self-expanding nitinol segment, and to study its effect on the von Mises stresses in the tram area.

### Mesh Sensitivity evaluation

The mesh of the stent was chosen in such a way to obtain relatively accurate results in a reasonable timeframe, resulting in indicative stress/strain values. The purpose of the FEA analysis was to show that the TRAM stent was effectively reopening the main branch, and does not preclude the possibility of a subsequent side branch stenting. Thus, the focus of the study was primarily the 'shape' of the vessel after stenting. Finite element analyses of coronary stenting have been mostly reported, and in the recent publications, the mesh sensitivity analysis is frequently replaced by the reference to previously published data.

Migliavacca et al. reported that for the finite element analysis of coronary artery stenting a mesh of 9000 elements was sufficient to obtain mesh-independent values of the von-Mises stress in the vessel [47]. In this study we used a mesh of 16470 hexahedral elements in the study. Moreover, the displacement analysis (i.e. the shape) is much less demanding than a stress-strain analysis with respect to mesh element resolution. Based on these considerations we can safely state that the chosen mesh resolution is above the minimal requirements for mesh independence.

## Results

### Stent and deployment characteristics

The stent was crimped after laser cutting and shape setting; and was deployed in the coronary artery bifurcation model over a balloon. The crimping profile of the stent was 1.0 mm. The stent deployed well in the coronary artery, and the tram area of the stent was well apposed to the sidewall. There was no distortion in the main vessel, in the side branch or the distal vessel. When the stent was deployed in the area with the lesion in the main branch, the stent opened well. The nitinol interface was positioned at the origin of the side-branch of the coronary artery. The tram area of the stent was overstretched marginally as the nitinol portion had higher target diameter than the proximal and distal cobalt chromium portion. The side-branch was well preserved, and the von-Mises stress was marginally higher at the nitinol portions of the stent.

### Mises stress and stress on the stent in phase 1 analysis

Though the von Mises stress was high, it was less compared to the currently existing methods of bifurcation treatment. The stress exerted on the stent by the coronary vessel was higher at the joints or the connections as shown in the Fig 2, and the stress on the stent overall was much lesser. The principal log strain was greater at the joints (see S1 and S2 Figs).

### Stage 2 analyses, and double stenting at side branch ostium

**Fig 5:**
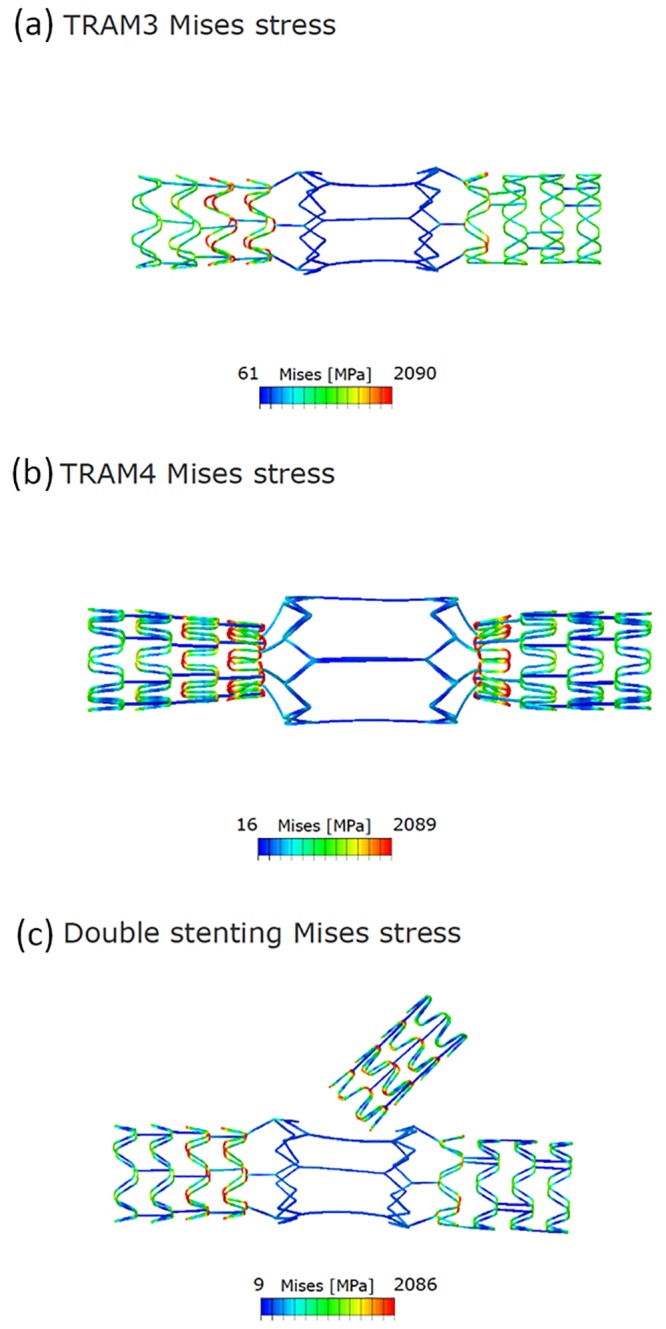
Finite element evaluation showing (a) Stress on the stent with 3 connecting links (b) Stress on the stent with 4 connecting links (c) Stress on the stent after double stenting.

**Fig 6:**
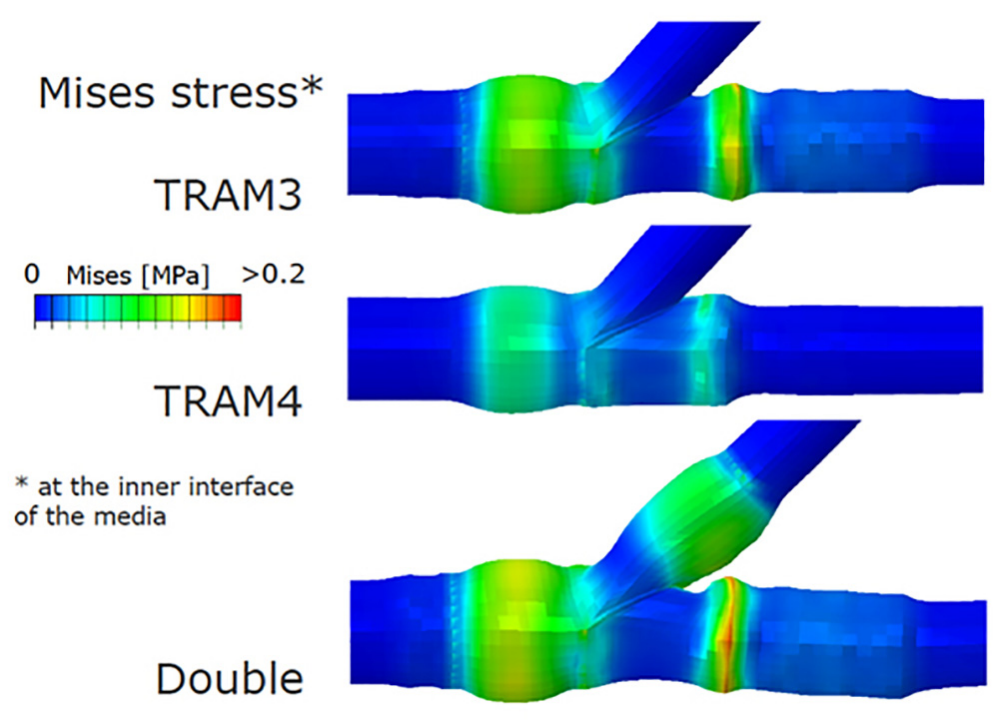
The results of the von Mises Stress on the coronary wall after deployment of the stent with 3 and 4 connecting links and after double stenting.

The contact pressures of the stent, and the tram areas of the stent are shown in the Fig 5. The nitinol part of the stent, which was the focus of attention in our evaluation, was seen as load bearing with high von-Mises stress (Fig 2). However, when the load bearing was tested with the stent dimensions reduced from 3.75 mm to 3.5 mm the stresses on the stent by the recoil effect of the arterial wall was marginally reduced as shown in the Fig 6. When the side branch was stented at the ostium the stresses increased in the distal part of the stent as shown in the illustration (Fig 6). The details of the principal log strain observed in the study are shown in S1, S2 and S3 Figs.

## Discussion

### Tram technique and Side Branch Stenting

The study has shown the potential possibilities of this new technique for the treatment of bifurcation lesions. The nitinol part serves as a connection loop in the form of a tram. This also provided space and support in the side-branch area. The side branch stenting of the tram method is the technique of placement of the side branch stent at the origin of the side-branch to reduce the metal load on the main vessel, which is essentially presumed to lessen the risk of thrombosis or restenosis in the bifurcation stents. The bifurcation model was built in such a way that it also could represent left main and its bifurcation branches with lesions. This method simplifies the bifurcation stenting procedure as this technique requires lesser balloons and avoids difficult re-crossing of the side-branches.

### Principal log strain and Von-Mises Stresses

Finite element studies of the currently available conventional bifurcation stenting methods have shown high peak stress and strain levels at the bifurcation after final kissing balloon or even after sequential stenting, with associated risk of fatigue fracture [11, 29, 48]. Conversely, in our study we obtained low value of stress and strain in the bifurcation region (peak strain < 0.03, see S1 and S3 Figs). Despite the geometrical complexity of the bifurcation region, the peak strain is similar to strain reported for straight regions [48,49]. This result can be ascribed to low metal mass of the nitinol connection segment at the bifurcation. The stresses in the arterial wall were higher near the Nitinol rings and reached a peak of 0.17MPa at the distal side, where vessel diameter was smaller, whereas it was below 0.06MPa in the other regions. These values reflect the results reported in other studies with a single Nitinol stent in a non-bifurcating vessel [48,49]. Hence, we speculate that the restenosis could even be lesser due to the smaller stress and strain in comparison to single stent deployed model. After deployment of the side-branch stent in our study the stresses in the side branch were in the range of 0.07 to 0.09MPa, which is again lesser than the stresses in the side branch in simultaneous kissing stenting 0.17 to 0.2MPa [29].

Also, when comparison of the stresses was performed with 3 and 4 connection struts, we have observed that the stress levels are significantly higher with 3 struts. Though 4 struts in tram offer strategically advantage, the manufacturing of the 4-connection links method is practically very difficult. Hence, the 4-connections in the tram area is not advisable. The modification from 3 to 4 strut was difficult, and it was achieved with unrealistic settings in strut-to-strut contact in the main area. FEA was performed after the amendment in the stent. The other alternative solution could be to mildly increase the thickness of the connection links to increase load bearing in the tram area.

The side branch stent was placed in the ostium of the side branch. This would be a unique method of stenting of the side branch. This is primarily to reduce the metal load in the stent and also to reduce balloon manipulations in the main branch, which could eventually cause stent fractures or stent distortions.

### Restenosis and Atherosclerosis

This method has less metal load at the bifurcation location and thereby lesser chances of restenosis [17]. Moreover, for the same reasons the chances of carinal shift also would be lesser. Significant dynamic changes happen in the side branch and distal part of the stent [50]. The drug deposition pattern in the arterial wall after drug eluting stents is determined by the luminal flow, which again is subject to variations in metal load and thereby the atherosclerotic and re-stenosis process [51, 52]. Therefore, this method has potential to offer a greater perspective for the future, though it is in the initial stages of evaluation.

Previous study has shown stent distortions after side branch ballooning irrespective of the stent dimensions [30]. Higher stress levels in the wall would lead to more endothelial proliferation and accelerate the atherosclerotic process [53–54]. Therefore, reducing the arterial wall stress would significantly help in reducing restenosis.

At this stage it is crucial to compare to existing treatment options (e.g. bifurcation stenting). The focus of this study was to introduce a new treatment option and explore its feasibility. The paper shows that the stenting system is effective in reopening the vessel and eases the introduction of additional stent is the side branch. Comparison to existing treatment is not within the scope of this study, as a fare comparison would require further optimization and finer modification of the stent design and procedure. However, the chosen bifurcation represents a realistic bench-test model, similar to bifurcation models used in recently published work [38].

### Simultaneous kissing (SKS) balloon techniques

Simultaneous kissing (SKS) balloon techniques have high-stress or strain levels in the stented coronary walls. Stent deployment in our study showed the wall strain (see S1, S2 and S3 Figs) were lower than the SKS or the sequential balloon techniques [29, 55], though the current study was not primarily focused for comparison of stresses or strains of other techniques. In the study by Foin et al. [55], the finite element assumptions and boundary conditions were similar to the present study. The strain observed in that study [55] was much higher than the strain observed in our study (see S1, S2 and S3 Figs). The primary advantage of this tram technique is the single stent method, and if required the side branch ostium could be stented easily. Also, the side branch stent can be placed at the ostium with minimum efforts. The stent can be positioned at any angle of bifurcation. Also, it could be used in any location of the bifurcation, and importantly its use could be extended in the setting myocardial infarction where the procedural time and contrast load is a crucial concern. In comparison to the dedicated stents for coronary bifurcations, which requires technical expertise and accurate placement techniques, this method is simpler and could be easily performed, and side branch stenting is easier. There are many methods of coronary bifurcation therapy. All Nordic studies report high complication rates with these procedures irrespective of the procedural techniques [26]. Therefore, at present single stent technique is the procedure of choice, and it is widely preferred and this tram method would help in the treatment of bifurcation lesions. Hence, this tram stent method would offer a novel strategy of stenting bifurcation lesions. However, further studies need to be performed in animal models for assessment of this tram stent technique. Computational flow dynamics could give further details of the flow pattern in the stent though the reduction in the wall stress in a well-deployed stent with a firm lesion is likely to be successful in the computational flow analysis also.

### Dedicated Bifurcation stents

Coronary dedicated bifurcation stents are available which are used for these types of lesions. These stents are technically demanding and require accurate placement of the stent in the lesion, and are they are more expensive and time consuming during deployment [56,57]. Though many stent methods are commercially available for this purpose the basic difficulty is in unpredictability of the coronary anatomy of the main vessel, and its marked variations in bifurcation. This is further complicated by the presence of ostial or osteo-proximal lesions in the side-branch. The origin of side-branch varies in each patient and the 3-Dimensional spatial orientation of the origin of the side-branch is not very clearly visualized in catheterization lab. Very few reference catheterization laboratories have 3-Dimensional visualization facilities, and most laboratories do not have this facility. For the same reasons, the placement of the dedicated stents is also difficult especially for the young interventionists, and hence in routine clinical practice most dedicated stents are not frequently used. Furthermore, most devices require additional shaft to be placed in the side branch of bifurcation lesion. Hence, we suppose this tram technique will circumvent many of the spatio-anatomical and deployment difficulties.

## Conclusion

There is potential for a novel technique in the treatment of bifurcation lesions. However, further studies need to be performed to further evaluate the treatment method *in-vivo* and *in-vitro*.

## Supporting Information

S1 Table Material properties of Nitinol. (TIF)

S1 Fig Details of the principal log strain observed in the study. (TIF)

S2 Fig Details of the principal log strain observed in the study with Tram 4 connections. (TIF)

S3 Fig Details of the principal log strain observed in the study with Tram 3 connections and side-branch stenting. (TIF)

## References

[1] Russell M, Binyamin G, Konstantino E. Ex vivo analysis of human coronary bifurcation anatomy: defining the main vessel-to-side-branch transition zone. EuroIntervention. 2009; 5(1):96–103. 19577989 10.4244/eijv5i1a15

[2] Athappan G, Ponniah T, Jeyaseelan L. True coronary bifurcation lesions: meta-analysis and review of literature. Journal of Cardiovascular Medicine. 2010; 11(2):103–110. 10.2459/JCM.0b013e32832ffc85 19952947

[3] Russell M, Binyamin G, Konstantino E. Ex vivo analysis of human coronary bifurcation anatomy: defining the main vessel-to-side-branch transition zone. EuroIntervention. 2009; 5(1): 96–103. 19577989 10.4244/eijv5i1a15

[4] Medina A, Suárez de Lezo J, Pan M. A New Classification of Coronary Bifurcation Lesions. Revista Española de Cardiología (English Edition). 2006; 59(2): 183. 16540043

[5] Sharma S, Sweeny J, Kini A. Coronary Bifurcation Lesions: A Current Update. Cardiology Clinics. 2010; 28(1): 55–70. 10.1016/j.ccl.2009.10.001 19962049

[6] Lefèvre T, Louvard Y, Morice MC. Stenting of bifurcation lesions: classification, treatments, and results. Catheter Cardiovasc Interv. 2000; 49: 274–283. 10700058 10.1002/(sici)1522-726x(200003)49:3<274::aid-ccd11>3.0.co;2-n

[7] Sgueglia G, Chevalier B. Kissing Balloon Inflation in Percutaneous Coronary Interventions. JACC: Cardiovasc Interv. 2012; 5(8): 803–811. 22917451 10.1016/j.jcin.2012.06.005

[8] Louvard Y. Percutaneous coronary intervention for bifurcation coronary disease. Heart. 2004; 90(6): 713–722. 15145893 10.1136/hrt.2002.007682 PMC1768265

[9] Sgueglia G, Todaro D, Bisciglia A, Conte M, Stipo A, Pucci E. Kissing inflation is feasible with all second-generation drug-eluting balloons. Cardiovascular Revascularization Medicine. 2011; 12(5): 280–285. 10.1016/j.carrev.2010.12.001 21273144

[10] Yoshimachi F., Masutani M., Matsukage T., Saito S., Ikari Y. Kissing balloon technique within a 5 Fr guiding catheter using 0.010 inch guidewires and 0.010 inch guidewire-compatible balloons. J Invasive Cardiol. 2007; 19(12): 519–24. 18180523

[11] Foin N, Torii R, Mortier P, De Beule M, Viceconte N, Chan P, et al Kissing Balloon or Sequential Dilation of the Side Branch and Main Vessel for Provisional Stenting of Bifurcations. JACC: Cardiovasc Interv. 2012; 5(1): 47–56. 22230150 10.1016/j.jcin.2011.08.019

[12] Heijer P, Bernink P, Van Dijk R, Twisk S, Lie K. The kissing balloon technique with two over-the-wire balloon catheters through a single 8-french guiding catheter. Cathet Cardiovasc Diagn. 1991;23 (1): 47–49. 1863962 10.1002/ccd.1810230113

[13] Ormiston J, Webster M, Webber B, Stewart J, Ruygrok P, Hatrick R. The “Crush” Technique for Coronary Artery Bifurcation Stenting: Insights From Micro-Computed Tomographic Imaging of Bench Deployments. JACC: Cardiovasc Interv. 2008; 1(4): 351–357. 19463329 10.1016/j.jcin.2008.06.003

[14] Guerin P, Pilet P, Finet G, Goueffic Y, N'Guyen J, Crochet D, et al Drug-Eluting Stents in Bifurcations: Bench Study of Strut Deformation and Coating Lesions. Circulation: Cardiovasc Interv. 2010; 3(2): 120–126. 10.1161/CIRCINTERVENTIONS.108.846089 20197512

[15] Chen S, Mintz G, Kan J, Zhang J, Hu Z, Ye F, et al Serial intravascular ultrasound analysis comparing double kissing and classical crush stenting for coronary bifurcation lesions. Cathet Cardiovasc Intervent. 2011; 78(5): 729–736. 10.1002/ccd.23110 21538789

[16] Farooq V, Serruys P, Heo J, Gogas B, Okamura T, Gomez-Lara J, et al New Insights Into the Coronary Artery Bifurcation. JACC: Cardiovasc Interv. 2011; 4(8): 921–931. 21851908 10.1016/j.jcin.2011.06.004

[17] Mortier P, Hikichi Y, Foin N, De Santis G, Segers P, Verhegghe B, et al Provisional Stenting of Coronary Bifurcations. JACC: Cardiovasc Interv. 2014; 7(3): 325–333. 24650404 10.1016/j.jcin.2013.09.012

[18] Morlacchi S, Colleoni S, Cárdenes R, Chiastra C, Diez J, Larrabide I, et al Patient-specific simulations of stenting procedures in coronary bifurcations: Two clinical cases. Med Eng Phys. 2013; 35(9): 1272–1281. 10.1016/j.medengphy.2013.01.007 23428836

[19] Kim B, Hong M, Shin D, Kim J, Ko Y, Choi D, et al Relationship between Stent Malapposition and Incomplete Neointimal Coverage after Drug-Eluting Stent Implantation. J Interv Cardiol. 2012; 25(3): 270–277. 10.1111/j.1540-8183.2011.00706.x 22372890

[20] Lassen J, Holm N, Stankovic G, Lefèvre T, Chieffo A, Hildick-Smith D, et al Percutaneous coronary intervention for coronary bifurcation disease: consensus from the first 10 years of the European Bifurcation Club meetings. EuroIntervention. 2014;10(5):545–560. 10.4244/EIJV10I5A97 25256198

[21] Steigen T, Maeng M, Wiseth R, Erglis A, Kumsars I, Narbute I, et al Randomized Study on Simple Versus Complex Stenting of Coronary Artery Bifurcation Lesions: The Nordic Bifurcation Study. Circulation. 2006; 114(18):1955–1961. 17060387 10.1161/CIRCULATIONAHA.106.664920

[22] Jensen J, Galløe A, Lassen J, Erglis A, Kumsars I, Steigen T, et al Safety in simple versus complex stenting of coronary artery bifurcation lesions. The Nordic Bifurcation Study 14-month follow-up results. EuroIntervention. 2008; 4(2):229–233. 19110788 10.4244/eijv4i2a41

[23] Hildick-Smith D, de Belder A, Cooter N, Curzen N, Clayton T, Oldroyd K, et al Randomized Trial of Simple Versus Complex Drug-Eluting Stenting for Bifurcation Lesions: The British Bifurcation Coronary Study: Old, New, and Evolving Strategies. Circulation. 2010; 121(10):1235–1243. 10.1161/CIRCULATIONAHA.109.888297 20194880

[24] Latib A, Colombo A, Sangiorgi G. Bifurcation stenting: current strategies and new devices. Heart. 2008;95(6):495–504. 10.1136/hrt.2008.150391 18812408

[25] Zhang F, Dong L, Ge J. Simple versus complex stenting strategy for coronary artery bifurcation lesions in the drug-eluting stent era: a meta-analysis of randomised trials. Heart. 2009; 95(20):1676–1681. 10.1136/hrt.2009.168641 19643768

[26] Behan M, Holm N, Curzen N, Erglis A, Stables R, de Belder A, et al Simple or Complex Stenting for Bifurcation Coronary Lesions: A Patient-Level Pooled-Analysis of the Nordic Bifurcation Study and the British Bifurcation Coronary Study. Circulation: Cardiovasc Interv. 2011; 4(1): 57–64. 10.1161/CIRCINTERVENTIONS.110.958512 21205942

[27] Katritsis D, Theodorakakos A, Pantos I, Gavaises M, Karcanias N, Efstathopoulos E. Flow Patterns at Stented Coronary Bifurcations: Computational Fluid Dynamics Analysis. Circulation: Cardiovasc Interv. 2012; 5(4): 530–539. 10.1161/CIRCINTERVENTIONS.112.968347 22763345

[28] Ormiston J, Webster M, Webber B, Stewart J, Ruygrok P, Hatrick R. The “Crush” Technique for Coronary Artery Bifurcation Stenting: Insights From Micro-Computed Tomographic Imaging of Bench Deployments. JACC: Cardiovasc Interv. 2008;1(4): 351–357. 19463329 10.1016/j.jcin.2008.06.003

[29] Mortier P, De Beule M, Dubini G, Hikichi Y, Murasato Y, Ormiston J. Coronary bifurcation stenting: insights from in vitro and virtual bench testing. EuroIntervention. 2010; 6(J): J53–J60. 21930491 10.4244/EIJV6SUPJA9

[30] Ormiston J, Webster M, Ruygrok P, Stewart J, White H, Scott D. Stent deformation following simulated side‐branch dilatation: A comparison of five stent designs. Cathet Cardiovasc Intervent. 1999; 47(2): 258–264. 10.1002/(SICI)1522-726X(199906)47:2<258::AID-CCD27>3.0.CO;2-C 10376516

[31] Prasad N, Seidelin PH. Sidebranch compromise during percutaneous coronary interventions. J Invasive Cardiol. 2002; 14:138–145. 11870269

[32] Finet G, Gilard M, Perrenot B, Rioufol G, Motreff P, Gavit L, et al Fractal geometry of arterial coronary bifurcations: a quantitative coronary angiography and intravascular ultrasound analysis. EuroIntervention. 2008; 3(4): 490–498. 19736093 10.4244/eijv3i4a87

[33] Martin D, Boyle F. Finite element analysis of balloon-expandable coronary stent deployment: Influence of angioplasty balloon configuration. Int J Numer Meth Biomed Engng. 2013; 29(11):1161–1175. 10.1002/cnm.2557 23696255

[34] Mortier P, De Beule M, Van Loo D, Verhegghe B, Verdonck P. Finite element analysis of side branch access during bifurcation stenting. Med Eng Phys. 2009; 31(4): 434–440. 10.1016/j.medengphy.2008.11.013 19117790

[35] Clerc C, Jedwab M, Mayer D, Thompson P, Stinson J. Assessment of wrought ASTM F1058 cobalt alloy properties for permanent surgical implants. Journal of Biomedical Materials Research. 1997; 38 (3): 229–234. 9283968 10.1002/(sici)1097-4636(199723)38:3<229::aid-jbm7>3.0.co;2-r

[36] P. Gong and A. R. Pelton. Finite element analysis on nitinol medical applications in SMST-2003: proceedings of the international conference on shape memory and superelastic technologies. 5 May to 8 May 2003, Asilomar Conference Center, Pacific1 Grove, California, USA, 2004, p. 443.

[37] Iannaccone F, De Bock S, De Beule M, Vermassen F, Van Herzeele I, Verdonck P, et al Feasibility of a priori numerical assessment of plaque scaffolding after carotid artery stenting in clinical routine: proof of concept. Int J Artif Organs. 2014; 37 (12): 928–39. 10.5301/ijao.5000379 25588766

[38] Auricchio F, Taylor R, Lubliner J. Shape-memory alloys: macromodelling and numerical simulations of the superelastic behavior. Comput Methods Appl Mech Eng. 1997; 146(3–4): 281–312.

[39] Holzapfel G. Determination of layer-specific mechanical properties of human coronary arteries with nonatherosclerotic intimal thickening and related constitutive modeling. Am J Physiol: Heart Circ Physiol. 2005; 289(5): H2048–H2058. 16006541 10.1152/ajpheart.00934.2004

[40] Mortier P, Hikichi Y, Foin N, De Santis G, Segers P, Verhegghe B, et al Provisional stenting of coronary bifurcations: insights into final kissing balloon post-dilation and stent design by computational modeling. JACC Cardiovasc Interv, 2014; 7 (3): 325–33. 10.1016/j.jcin.2013.09.012 24650404

[41] Loree HM, Grodzinsky AJ, Park SY, Gibson LJ, Lee RT. Static circumferential tangential modulus of human atherosclerotic tissue. J Biomech. 1994; 27(2): 195–204. 8132688 10.1016/0021-9290(94)90209-7

[42] De Santis G, Mortier P, De Beule M, Segers P, Verdonck P, Verhegghe B. Patient-specificcomputational fluid dynamics: structured mesh generation from coronary angiography. Med Biol Eng Comput, 2010; 48 (4): 371–80. 10.1007/s11517-010-0583-4 20162466

[43] De Bock S, Iannaccone F, De Santis G, De Beule M, Van Loo D, Devos D, et al Virtual evaluation of stent graft deployment: a validated modeling and simulation study. J Mech Behav Biomed Mater. 2012; 13: 129–39. 10.1016/j.jmbbm.2012.04.021 22842656

[44] Mortier P, Holzapfel G, De Beule M, Van Loo D, Taeymans Y, Segers P, et al A Novel Simulation Strategy for Stent Insertion and Deployment in Curved Coronary Bifurcations: Comparison of Three Drug-Eluting Stents. Ann Biomed Eng. 2009; 38(1): 88–99. 10.1007/s10439-009-9836-5 19898936

[45] Conti M, Van Loo D, Auricchio F, De Beule M, De Santis G, Verhegghe B, et al Impact of carotid stent cell design on vessel scaffolding: a case study comparing experimental investigation and numerical simulations. J. Endovasc. Ther. 2011; 18(3): 397–406. 10.1583/10-3338.1 21679082

[46] Auricchio F, Conti M, De Beule M, De Santis G, Verhegghe B. Carotid artery stenting simulation: from patient-specific images to finite element analysis. Med Eng Phys 2011; 33(3): 281–9. 10.1016/j.medengphy.2010.10.011 21067964

[47] Migliavacca F, Gervaso F, Prosi M, Zunino P, Minisini S, Formaggia L, et al Expansion and drug elution model of a coronary stent. Comput Methods Biomech Biomed Engin. 2007; 10(1): 63–73. 10.1080/10255840601071087 18651272

[48] Zhao S, Gu L, Froemming S. Effects of arterial strain and stress in the prediction of restenosis risk: Computer modeling of stent trials. Biomed Eng Lett. 2012; 2(3):158–163.

[49] Zhao S, Gu L, Froemming S. Assessment of shape memory alloy stent deployment in a stenosed artery. Biomed Eng Lett. 2011;1(4):226–231.

[50] Williams Andrew R., Koo Bon-Kwon, Gundert Timothy J., Fitzgerald Peter J., LaDisa John F.. Local hemodynamic changes caused by main branch stent implantation and subsequent virtual side branch balloon angioplasty in a representative coronary bifurcation. J Appl Physiol. 2010; 109 (2): 532–540. 10.1152/japplphysiol.00086.2010 20507966

[51] Kolachalama VB, Levine EG, Edelman ER. Luminal flow amplifies stent-based drug deposition in arterial bifurcations. PLoS One. 4: e8105, 2009 10.1371/journal.pone.0008105 19956555 PMC2781163

[52] Kwak B, Bäck M, Bochaton-Piallat M, Caligiuri G, Daemen M, Davies P, et al Biomechanical factors in atherosclerosis: mechanisms and clinical implications. Eur Heart J. 2014; 35(43): 3013–3020. 10.1093/eurheartj/ehu353 25230814 PMC4810806

[53] Malek AM, Alper SL, Izumo S. Hemodynamic shear stress and its role in atherosclerosis. JAMA 1999; 282: 2035–2042. 10591386 10.1001/jama.282.21.2035

[54] Stone PH, Coskun AU, Kinlay S, Clark ME, Sonka M, Wahle A, et al Effect of endothelial shear stress on the progression of coronary artery disease, vascular remodeling, and in-stent restenosis in humans: in vivo 6-month follow-up study. Circulation 2003;108: 438–444. 12860915 10.1161/01.CIR.0000080882.35274.AD

[55] Foin N, Torii R, Mortier P, DeBeule M, Nicola V, Pak HC, et al Kissing Balloon or Sequential Dilation of the Side Branch and Main Vessel for Provisional Stenting of Bifurcations: Lessons From Micro-Computed Tomography and Computational Simulations. J Am Coll Cardiol Intv. 2012; 5(1): 47–56. 10.1016/j.jcin.2011.08.019 22230150

[56] Pillai A, Jayaraman B. Dedicated bifurcation stents. Indian Heart Journal. 2012; 64(2):187–195. 10.1016/S0019-4832(12)60059-5 22572498 PMC3860958

[57] Waksman R, Bonello L. The 5 Ts of Bifurcation Intervention: Type, Technique, Two Stents, T-Stenting, Trials, Editorials published in JACC: Cardiovascular Interventions reflect the views of the authors and do not necessarily represent the views of JACC: Cardiovascular Interventions or the American College of Cardiology. JACC: Cardiovasc Interv. 2008; 1(4):366–368. 19463331 10.1016/j.jcin.2008.06.006

