# Supplementary figures and images for: A Novel Tram Stent Method in the Treatment of Coronary Bifurcation Lesions – Finite Element Study
Source: PLoS One. 2016 Mar 3;11(3):e0149838. doi: 10.1371/journal.pone.0149838 (PMC4777498; doi:10.1371/journal.pone.0149838)

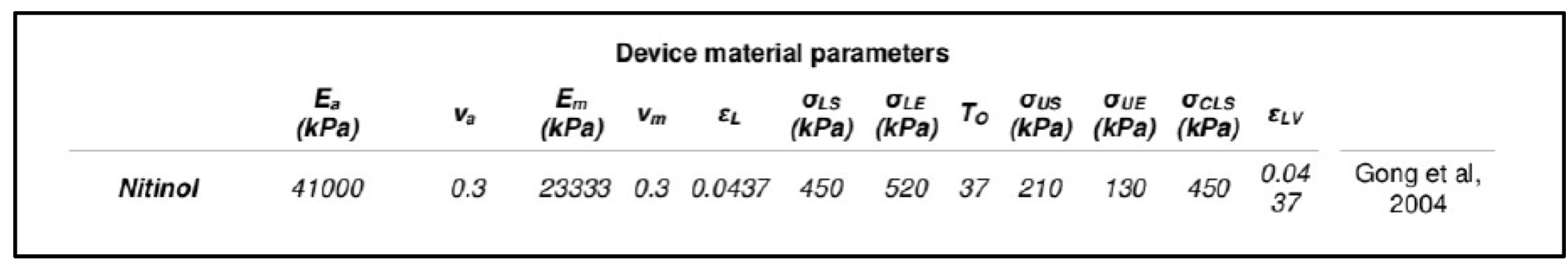

Supplement: S1 Table — (TIF) [file pone.0149838.s001.tif]

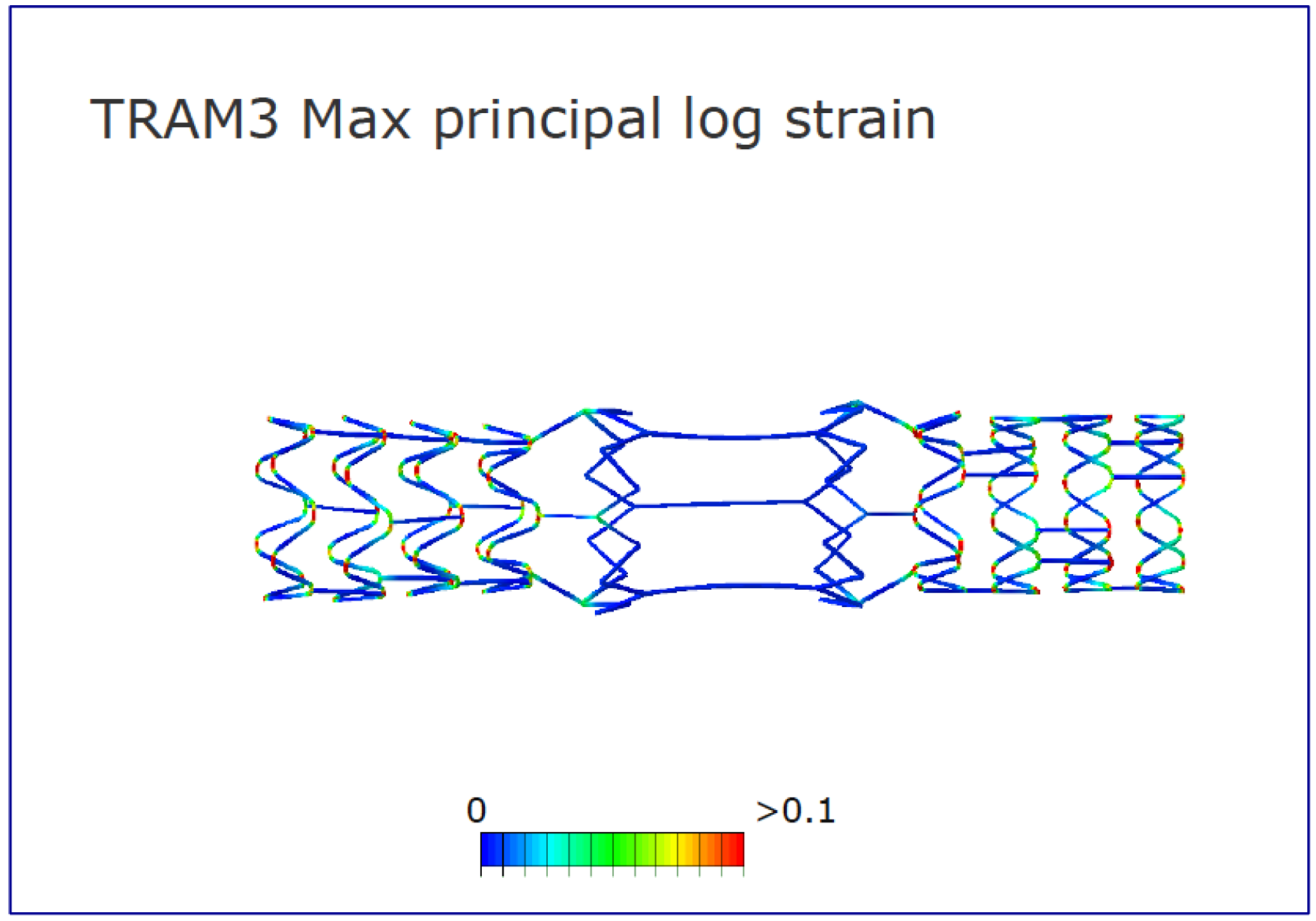

Supplement: S1 Fig — (TIF) [file pone.0149838.s002.tif]

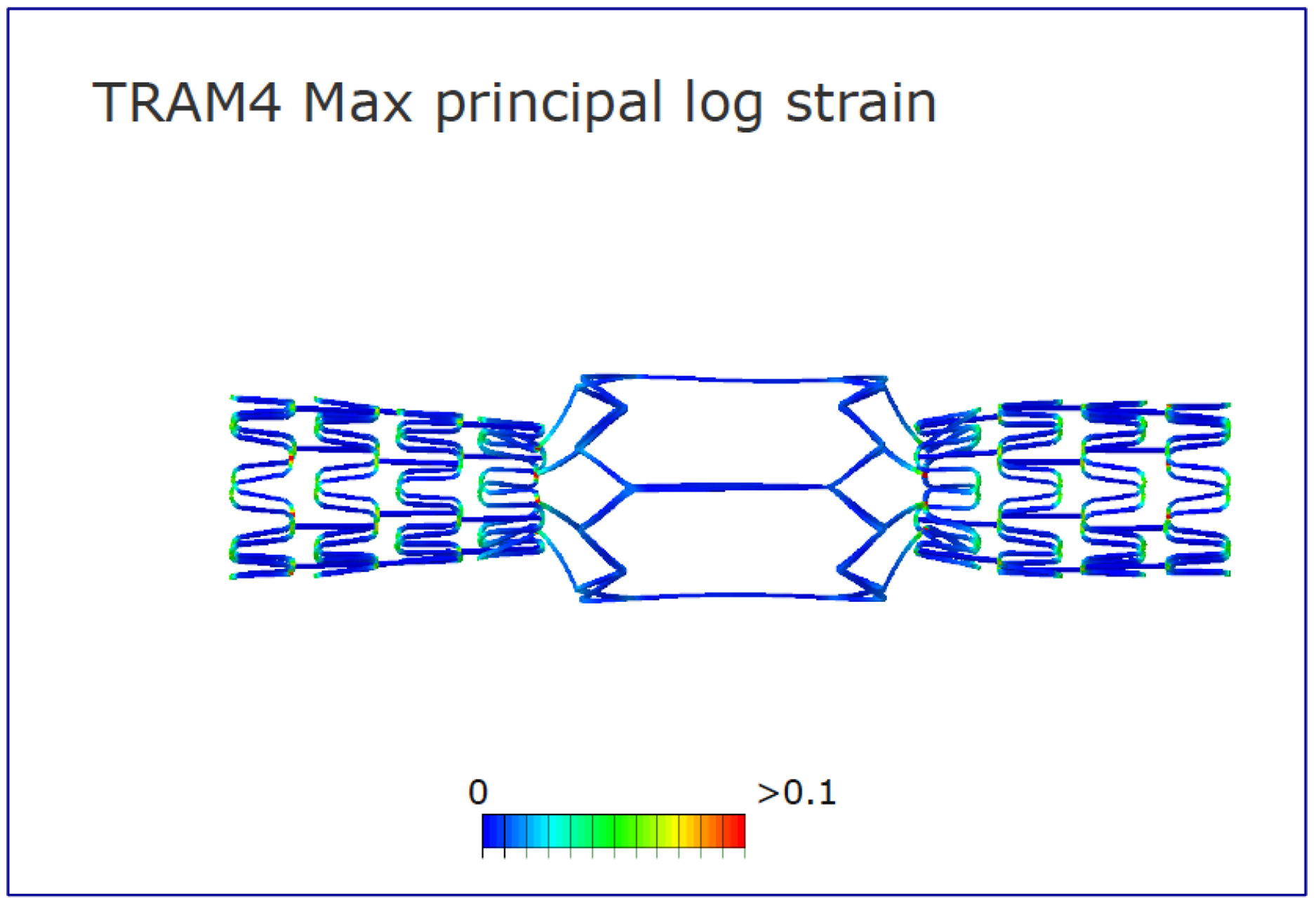

Supplement: S2 Fig — (TIF) [file pone.0149838.s003.tif]

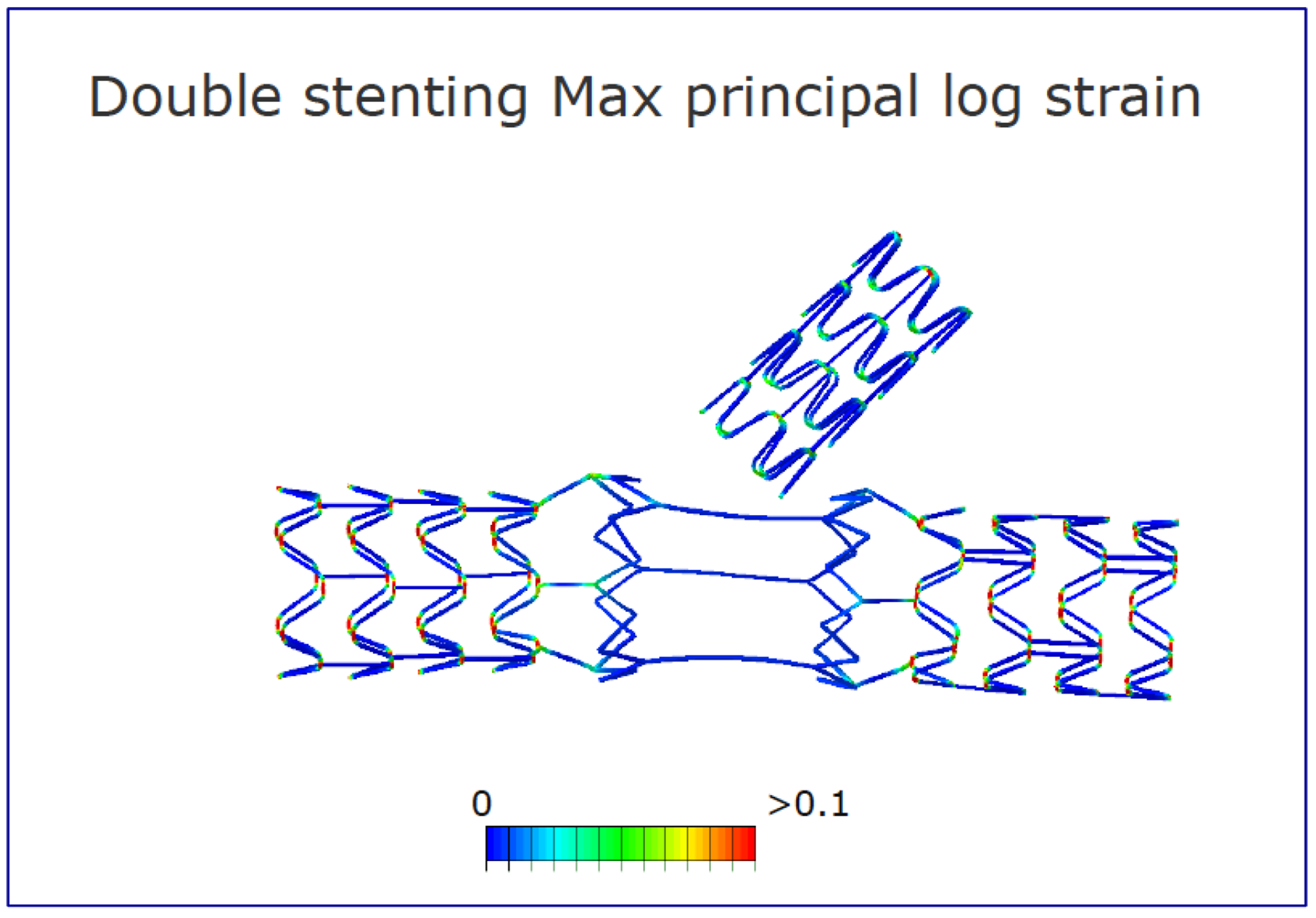

Supplement: S3 Fig — (TIF) [file pone.0149838.s004.tif]
